# Syngas obtained by microwave pyrolysis of household wastes as feedstock for polyhydroxyalkanoate production in *Rhodospirillum rubrum*

**DOI:** 10.1111/1751-7915.12411

**Published:** 2016-09-28

**Authors:** Olga Revelles, Daniel Beneroso, J. Angel Menéndez, Ana Arenillas, J. Luis García, M. Auxiliadora Prieto

**Affiliations:** ^1^ Centro de Investigaciones Biológicas, CSIC C/ Ramiro de Maeztu, 9 28040 Madrid Spain; ^2^ Instituto Nacional del Carbón, CSIC Apartado 73 33080 Oviedo Spain; ^3^ Present address: Microwave Process Engineering Research Group Faculty of Engineering The University of Nottingham Nottingham NG7 2RD UK

## Abstract

The massive production of urban and agricultural wastes has promoted a clear need for alternative processes of disposal and waste management. The potential use of municipal solid wastes (MSW) as feedstock for the production of polyhydroxyalkanoates (PHA) by a process known as syngas fermentation is considered herein as an attractive bio‐economic strategy to reduce these wastes. In this work, we have evaluated the potential of *Rhodospirillum rubrum* as microbial cell factory for the synthesis of PHA from syngas produced by microwave pyrolysis of the MSW organic fraction from a European city (Seville). Growth rate, uptake rate, biomass yield and PHA production from syngas in *R. rubrum* have been analysed. The results revealed the strong robustness of this syngas fermentation where the purity of the syngas is not a critical constraint for PHA production. Microwave‐induced pyrolysis is a tangible alternative to standard pyrolysis, because it can reduce cost in terms of energy and time as well as increase syngas production, providing a satisfactory PHA yield.

## Introduction

The development of a bio‐based industry requires the production of chemicals and biomaterials to be settled on the exploitation of non‐fossil carbon resources. Municipal solid wastes (MSW) and other organic waste resources, such as agricultural residues and sewage sludge contain significant reusable carbon fractions suitable for eco‐efficient valorization processes. These resources take advantage over fossil sources as they are abundantly available, do not require additional production costs and are substrates that do not compete with human nutrition chain.

**Figure 1:**
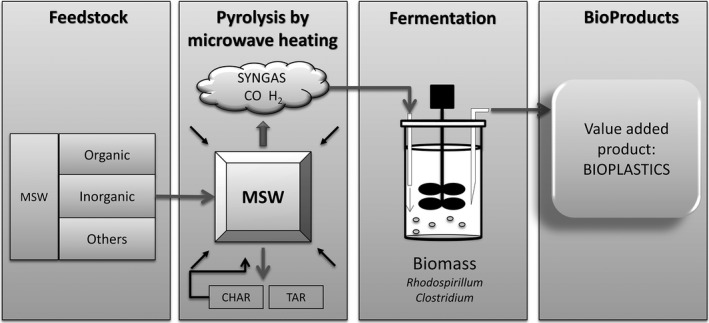
Sustainable bioconversion of MSW into PHB process. MSW from Seville landfill were collected and subjected to MIP‐induced pyrolysis in a microwave oven. The obtained syngas is further fermented by *R. rubrum*‐producing PHB.

In this context, thermochemical conversion technologies of wastes, other than combustion, such as pyrolysis and gasification are becoming widely accepted as suitable valorization alternatives for non‐fossil resources (Arena, 2012; Tanigaki *et al*., 2013). For instance, the gasification of organic wastes produces syngas (CO+H_2_) that can be used as chemical platform for the production of bulk chemicals such as ethanol, butanol, acetic acid or butyric acid (Munasinghe and Khanal, 2010). In this regard, the microbial bioconversion of thermochemically processed wastes presents a highly attractive potential nowadays (Koutinas *et al*., 2014; Drzyzga *et al*., 2015) (Fig. 1). Of special interest is the development of the chemical industry devoted to produce bio‐based and biodegradable plastics, such as polyhydroxyalkanoates (PHA), with many applications including biomedical uses (Reddy *et al*., 2003; Chen and Wu, 2005; Madbouly *et al*., 2014; Drzyzga *et al*., 2015). These bioplastics represent an alternative to oil‐derived plastics and are degraded by many microorganisms, having gained importance in the last years (Keshavarz and Roy, 2010; Nikodinovic‐Runic *et al*., 2013).

The production of syngas from biomass and organic wastes is mainly accomplished by means of gasification processes (Mahinpey and Gomez, 2016), although new emerging thermal conversion technologies, such as the microwave‐induced pyrolysis (MIP) has been reported as an effective and greener way to increase the waste conversion to H_2_ and CO (Budarin *et al*., 2011; Beneroso *et al*., 2013, 2014, 2015a,b,c). The attractiveness behind MIP is the fact that microwaves are able to induce an instantaneous and volumetric heating within the samples and thus, overcoming heat transfer constraints as a result of the low thermal conductivity of biomass and biowastes (Beneroso *et al*., 2015b; Beneroso and Fidalgo, 2016a). In addition to syngas and tars, a carbonaceous solid fraction (known as char) is produced during MIP. This fraction has a low value owing to its high ash content, although this can be used as a solid fuel or for soil amendment purposes. Beneroso *et al*. (2016b) has recently reported the potential use of char as an additive to soils, and particularly, the char obtained from MSW can be added to soil for carbon sequestration without negatively affecting the soil fertility. Therefore, MIP is an attractive alternative within thermochemical technologies for syngas production (Beneroso *et al*., 2015b).

Despite the toxicity of CO for most organisms, several microorganisms can use CO as source of carbon and/or energy for growth. *Rhodospirillum rubrum*, which is the type strain for the *Rhodospirillaceae* family, is able to grow under a broad variety of conditions, including syngas (Do *et al*., 2007). This bacterium can utilize CO under anaerobic conditions as a sole carbon and energy source in the presence or absence of light (Kerby *et al*., 1995; Revelles *et al*., 2016a,b). It is important to emphasize that *R. rubrum* has a photosynthetic apparatus involved in the photosynthesis on anaerobic conditions. When exposed to CO, both a CODH and a CO‐insensitive hydrogenase are induced catalysing the oxidation of CO into CO_2_ and H_2_. Approximately, 40% of the carbon fraction of syngas (CO_2_ and CO) is assimilated into biomass. Although *R. rubrum* is a highly versatile bacterium that possess the Calvin–Benson–Bassham cycle, it has been recently shown that other carboxylases than Rubisco are actively incorporating CO_2_ from syngas into biomass (Revelles *et al*., 2016a,b). Furthermore, *R. rubrum* is an attractive syngas utilizer for its ability to produce PHA as an energy storage molecule during the fermentation process. We recently reported around 20% of poly(3‐hydroxybutyrate) (PHB) production on this microorganism during syngas fermentation both in darkness and/or light (Revelles *et al*., 2016a,b). Other studies reported the heterologous overexpression of different genes encoding pyridine nucleotide transhydrogenases (pntAB, udhA) and acetoacetyl‐CoA reductases (PhaB) in *R. rubrum* S1, during the synthesis of the copolymer poly(3‐hydroxybutyrate‐*co*‐3‐hydroxyvalerate) P(HB‐HV) from syngas (Heinrich *et al*., 2015). This engineered bacterium was pointed out as a promising production strain for syngas‐derived, second‐generation biopolymers, increasing the potential of this strain for industry application.

The utilization of MSW‐derived syngas for the sustainable production of PHB is shown in this work. Of particular interest is the innovative use of MIP to achieve this goal, as this technology provides a syngas able to induce an efficient growth of *R. rubrum*, reducing cost in terms of energy and time at a much higher syngas productivity compared with syngas from conventional pyrolysis technologies.

## Results and discussion

### Growth of *R. rubrum* in microwaving versus synthetic syngas

An organic fraction from a MSW provided by ABENGOA BIOENERGÍA from a landfill in Seville (Spain) was used in this study. The MSW fraction was subjected to removal of moisture and inert solids, such as glass or metals. After this pre‐treatment, the fraction size was reduced to 1–3 mm. The characterization of the sample can be found somewhere else (Beneroso *et al*., 2015a). To prepare syngas, the waste was subjected to MIP in a microwave oven (Beneroso *et al*., 2015a). The char from previous pyrolysis experiments was used as microwave receptor to induce the pyrolysis due to the low capacity of organic wastes to absorb microwaves. When the microwaves pass through the sample, they are absorbed by the receptor and the temperature increases, allowing the heat to be conducted to the waste to reach a temperature high enough to start the pyrolysis. As the pyrolysis proceeds, the waste is carbonized and is then able to absorb microwaves, so that from that point on it can be directly heated by microwave radiation. The detailed methodology has been described previously (Beneroso *et al*., 2015a). Table 1 shows the syngas composition of the MIP compared with the standard synthetic syngas used for bacterial fermentation (Revelles *et al*., 2016a,b).

**Table 1:** Gas composition of syngas

Gases	Syngas composition (vol.%)^a^	
	Synthetic	MIP
CO	40	27
CO_2_	10	6
H_2_	40	37
N^b^_2_	10	26
CH_4_+C_2_H_4_+C_2_H_6_	0	4

a Synthetic syngas was provided by Air Liquide (Air Liquide, www.airliquide.com). Composition of microwaving syngas was determined in a gas chromatograph (GC, Agilent 7890A) equipped with a TCD and two columns connected in series (80/100 Porapak Q and 70/80 Molesieve 13X). The initial oven temperature was 30°C, which was maintained with an isothermic step of 5 min. It was then programmed with a rate of 25°C min^−1^ until reached 180°C. The injector and detector temperatures were 150 and 250°C respectively. Helium (Air Liquide, www.airliquide.com) was used as carrier gas. Prior to the measurements, the gas analyser was calibrated by a standard gas and a calibration curve was established. The calculation for gas concentration was carried out using the GC data analysis software (ChemStation rev. B.04.03‐SP1; Agilent Technologies, Santa Clara, CA 95051, United States).

b The synthetic gas was balanced with N_2_, while in the mixture of gases from MIP, the N_2_ content is due to the N_2_ used as carrier gas but not released in the pyrolysis process.

Starter cultures of *R. rubrum* (ATCC 11170) were grown under anaerobic conditions on SYN medium (Revelles *et al*., 2016aa) supplemented with 15 mM fructose at 30°C during 48 h until stationary phase (OD_600_ 1.5). This culture was used as pre‐inoculum for syngas fermentation.

Syngas experiments were done in bottles of 100 mL containing 20 mL of SYN medium supplemented with 10 mM acetate as we had previously demonstrated that the presence of acetate during syngas fermentation favours PHB accumulation (Revelles *et al*., 2016a,b). Before adding syngas, the closed degasified serum vials were subjected to 1 min vacuum‐purge and the atmosphere was further saturated with syngas, either synthetic or microwave‐derived syngas, to 1 atm of pressure. This procedure for syngas feeding was repeated every day.

*R. rubrum* is a highly versatile bacterium that can ferment syngas either in dark or in light. On anaerobic conditions when the cells are exposed to light, the photosynthesis becomes active being this an extra energy source. Therefore, the potential impact of light on cell physiology during syngas fermentation was also evaluated. A syngas sample obtained from the MIP process and the synthetic syngas were used as substrates for syngas fermentation in parallel cultures of SYN medium supplemented with 10 mM of acetate in darkness and in light. A very detailed protocol for culturing *R. rubrum* in syngas both in darkness and in light can be found in Revelles *et al*. (2016a,b).

Table 2 shows the fermentation kinetic parameters, such as growth rate, μ; acetate uptake rate, Qs; and biomass dry weight production yield. None difference in the growth rate μ was observed among the two different syngas tested in light or darkness, i.e., 0.030 h^−1^ and 0.022 h^−1^ respectively. In both syngas, a higher growth rate was detected in light. It is worthy to stress that light has a positive impact in the growth rate, likely due to the contribution of the photosynthesis in the energetic status of the cell. The small presence of hydrocarbons (methane, ethylene and ethane) within the MIP‐derived syngas (Table 1) does not have a harmful effect on bacterial growth. By high‐performance liquid chromatography analysis, the presence of extracellular products such as malate, succinate, propionate, pyruvate and/or formate were measured, but none of them were detected in the supernatant. Interestingly, when MIP‐derived syngas was used in the fermentation process, 1.5‐ to 2‐fold higher acetate uptake rate was found compared with synthetic syngas in light and darkness respectively (Table 2). This effect was observed in the yield of biomass from acetate as well, being 1.5‐fold higher in MIP‐derived syngas than in synthetic syngas (Table 2).

**Table 2:** Kinetic growth parameters of *R. rubrum* with syngas in light and darkness

Kinetic growth	Synthetic		MIP	
	Light	Darkness	Light	Darkness
μ (h^−1^)	0.029 ± 0.005	0.021 ± 0.005	0.031 ± 0.005	0.022 ± 0.005
Qs (mmol gDW^−1^ h^−1^)	1.51 ± 0.05	1.43 ± 0.05	2.05 ± 0.20	3.10 ± 0.15
Q_CO_ (mmol_CO_ gDW^−1^ h^−1^)	0.01	ND	0.005	ND
Y (gDW g^−1^)	0.23 ± 0.05	0.17 ± 0.01	0.25 ± 0.05	0.13 ± 0.05
PHB (% CDW)	20 ± 5	28 ± 10	16 ± 1	10 ± 1

Parameters: μ (h^−1^), specific growth rate; Q_s_ (mmol gDW^−1^ h^−1^), acetate uptake rate; Y (gDW g^−1^), biomass dry weight production yield; PHB (% cell dry weight). Values represent the mean ± standard deviation of three independent biological replicates. The growth rate (μ) was determined from log‐linear regression of time‐dependent changes in optical density at 600 nm (OD600), measured with a spectrophotometer (UV‐VIS Spectrophotometer Shimatzu UV mini 1240) with appropriate dilutions when needed. To calculate specific biomass yields, correlation factors between cell dry weights and optical density (gCDW/OD600) were established for each condition. Acetate disappearance was quantified using an high‐performance liquid chromatography system (GILSON), equipped with an Aminex HPX‐87H column and a mobile phase of 2.5 mM H_2_SO_4_ solution at a 0.6 mL min^−1^ flow rate operated at 40°C.

ND, non‐determined.

### CO consumption and PHB production during the syngas fermentation

CO assimilation in *R. rubrum* is associated to the CODH enzyme that catalyses the oxidation of CO into CO_2_. When the cells are growing on syngas plus acetate, the carbon fraction of syngas is incorporated into biomass and PHB by the activity of different carboxylases of the central carbon metabolism (Revelles *et al*., 2016a,b). Acetate assimilation involves two steps: the ferredoxin‐dependent pyruvate synthase (PFOR) enzyme and the ethylmalonyl‐CoA pathway (Revelles *et al*., 2016a,b). The use of acetate during syngas fermentation is critical to achieve PHB accumulation, where approximately 40% of PHB total carbon backbone comes from the carbon fraction of syngas and the remaining fraction comes from acetate (Revelles *et al*., 2016a,b). PHB is produced by a biosynthetic pathway consisting of three different enzymatic reactions from acetyl‐CoA. The first step is the condensation of two acetyl‐CoA molecules into acetoacetyl‐CoA by 3‐ketothiolase (catalyses by the enzyme PhaA). Then, acetoacetyl‐CoA reductase (PhaB) allows the reduction of acetoacetyl‐CoA by NADH to 3‐hydroxybutyryl‐CoA. Finally, the (*R*)‐3‐hydroxybutyryl‐CoA monomers are polymerized into PHB by PHB synthase (PhaC).

The CO consumption at the end of the growth curve for each different syngas fermentation process was monitored. As no difference was registered between light and darkness, the CO consumption was analysed on light. A sample (1 mL) from the head‐space of the culture bottle was taken at time zero and after 48 h of incubation in light. The differences in the final CO values were measured using a gas chromatograph equipped with a thermal conductivity detector (TCD). The results on CO consumption as percentage of gas converted and the uptake of CO are shown in Tables 2 and 3 respectively. The CO conversion registered was of about 40% in both synthetic and MIP‐derived syngas. Although in both cases the % of CO conversion does not differ, the final amount of metabolized CO is half for MIP syngas than synthetic syngas (Table 3, 0.19 versus 0.44 mmoles of CO respectively). The lower CO uptake (see Table 2) determined for MIP syngas is compensated with a higher acetate uptake (Qs) observed in this syngas to keep a constant growth rate.

**Table 3:** CO consumption during syngas fermentation

Gas	% Conversion^a^		mmol of CO^b^	
	Synthetic	MIP	Synthetic	MIP
CO	39 ± 4	37 ± 8	0.44 ± 0.05	0.19 ± 0.1

a The differences in the final gas composition are given as percentage of gas consumed.

b mmol of CO consumed at the end of the growth.

A gas chromatograph (GC, Agilent 7890A) was equipped with a thermal conductivity detector (TCD) and two columns connected in series (80/100 Porapak Q and 70/80 Molesieve 13X) as described for 1.

Values represent the mean ± SD of three independent biological replicates.

PHB production was quantified from cells harvested from cultures grown in syngas by centrifugation at 8000 *g* for 15 min at 4°C (Eppendorf Centrifuge 5810R). Cells were then washed twice in distilled water and lyophilized in Cryodos‐50 (Telstar, Terrasa, Spain) at −56°C and 10^‐2^ mbar. Furthermore, PHB monomer composition and cellular PHB content were determined by gas chromatography (GC) of the methanolysed polyester. Methanolysis was carried out by suspending 5–10 mg of lyophilized cells in 0.5 mL of chloroform and 2 mL of methanol containing 15% sulfuric acid and 0.5 mg mL^−1^ of 3‐methylbenzoic acid (internal standard), followed by an incubation at 80°C for 7 h. After cooling, 1 mL of demineralized water and 1 mL of chloroform were added and the organic phase containing the methyl esters was analysed as described previously (de Eugenio *et al*., 2010). A standard curve from 0.5 to 2 mg of PHB (Cat: 36,350‐2; Sigma, San Luis, Misuri, United States) was used to interpolate sample data.

**Figure 2:**
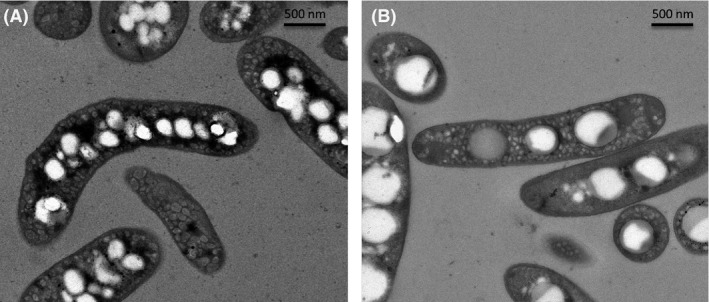
Transmission electron micrograph (TEM) of *R. rubrum* growing in medium SYN with acetate fed microwave‐induced pyrolysis syngas (A) and synthetic syngas (B) both containing PHB granules. Culture was harvested, washed twice in PBS and fixed in 5% (w/v) glutaraldehyde in the same solution. The cells were incubated with 2.5% (w/v) OsO_4_ for 1 h, gradually dehydrated in ethanol solutions and propylene oxide and embedded in Epon 812 resin. Ultrathin sections (50–70 nm) were cut and observed using a Jeol‐1230 electron microscope.

Table 2 shows the PHB content of cells of *R. rubrum* from MSW‐derived syngas versus synthetic syngas. When light was used as an extra source of energy, the percentage of PHB detected remained similar regardless of the source of syngas (16 ± 5% and 20 ± 1% respectively). But, a small reduction was detected in cells grown on darkness (28 ± 10% and 10 ± 1% respectively). Furthermore, granules of PHB can be clearly observed in *R. rubrum* grown in MIP‐derived syngas by transmission electronic microscopy during syngas fermentation when using MIP‐derived syngas (Fig. 2).

In this work, it has been shown that the use of wastes‐derived syngas did not affect the growth rate of *R. rubrum* being of ~0.02 μ^−1^, but an increase in the acetate uptake rate was found indicating a higher consumption of acetate that will compensate the lower CO consumption observed in MIP syngas, although CO conversion does not change. The lower CO consumption could be explained by the lower percentage of CO presence in MIP syngas. Interestingly, the accumulation of PHB observed in cells grown with MIP‐derived syngas was similar to that found on synthetic syngas, especially when they were exposed to light. These results are a proof of concept of the potential of pyrolysed MSW as feedstock for the production of bioplastics. Furthermore, the robustness and versatility of *R. rubrum* to accumulate PHB regardless of the composition of the syngas increases the industrial interest of this bacterium to be used in syngas fermentation without introducing a previous costly step for syngas purification. The capability of this microorganism to synthesize other polymers different from PHA of industrial interest from different carbon sources has been recently shown in an engineering strain (Heinrich *et al*., 2015) broadening its potential to be used in biopolymers production industry. In summary, this work represents the first experimental demonstration that MIP of MSW followed by syngas fermentation can become a valuable environmental‐friendly strategy for waste treatment rendering high value‐added products such as bioplastics.

## Conclusion

The MSW generation has boosted with the increase in economic growth and population as well as with the living standards of developing countries. Finding a solution to manage MSW is a complex issue that needs to assess different social aspects including environmental impact and economic cost. One of the main bottle necks of using MSW as feedstocks is the complexity of the organic fraction composition, which hinders its application in conventional bioprocesses using microbial monocultures. In this study, the potential use of MSW as feedstock for the production of PHB by *R. rubrum* has been demonstrated by subjecting MSW to an emerging thermochemical process based on microwave heating, which enables the production of syngas useful as a derived feedstock. The effects of using syngas as feedstock on the physiology of *R. rubrum* was investigated demonstrating the robustness of this bacterium as biocatalyst, as the growth, syngas consumption and PHB production are not affected by the putative contaminants present in MSW‐derived syngas. Our results expand the potential of this hybrid technology to be implemented in waste management programmes. Within this general strategy, the use of MIP provides an additional advantage versus conventional oven pyrolytic processes, because it reduces time and energy for syngas synthesis, hence reducing cost and permitting a much higher syngas volumetric productivity, without affecting syngas fermentation.

## Conflict of interest

The authors declare no conflict of interest.
